# Factors related to decreased bone mineral density in childhood cancer survivors

**DOI:** 10.1186/1687-9856-2013-S1-P156

**Published:** 2013-10-03

**Authors:** Yun Jung Choi, Moon Hee Lee, Won Kyoung Cho, Kyoung Soon Cho, So Hyun Park, Min Ho Jung, Byung Kyu Suh, Byung Churl Lee

**Affiliations:** 1 Pediatrics, College of Medicine, The Catholic University of Korea, Seoul, Republic of Korea

## 

The risk of osteoporosis or osteopenia is known to increase after childhood cancer treatment. The purpose of this study was to evaluate patterns of bone mineral density (BMD) and factors related to decreased BMD in childhood cancer survivors.

We studied 78 patients (34 males, 44 females) treated for acute lymphoblastic leukemia, acute myelogenous leukemia or chronic myelogenous leukemia during childhood. Clinical data, laboratory finding, bone age, lumbar BMD (LBMD) and femur neck BMD (FNBMD) were investigated. Chronological age at evaluation was 11.6±3.4 yr in males and 13.0±3.3 yr in females. Primary disease, chronological age at treatment, body mass index (BMI), method of treatment (chemotherapy, radiotherapy), endocrine function, presence of chronic graft-versus-host disease (cGVHD) and relevance of BMD standard deviation score (SDS) were evaluated. LBMD and FNBMD of the subjects were -0.91±1.41 and -1.13±1.79, respectively. Twenty (25.7%) patients had LBMD SDS lower than -2. Nineteen (24.4%) patients had FNBMD SDS lower than -2. The patients treated with hematopoietic stem cell transplantation had lower LBMD SDS (-1.17±1.39 vs -0.43±1.33, *P*=0.025). The risk of having LBMD SDS <-2 was higher in the patients treated with glucocorticoid for cGVHD (36.6% vs 13.5%; odds ratio [OR], 3.7; *P*=0.020). In multivariate logistic regression analysis, longer duration of glucocorticoid treatment (OR, 1.12; 95% confidence interval [CI], 1.03-1.22) and lower BMI SDS (OR, 0.42; 95% CI, 0.21-0.83) were associated with decreased LBMD SDS. These findings suggest that prolonged glucocorticoid use and reduction in BMI are risk factors for decreased BMD in childhood cancer survivors. Anticipatory follow-up and appropriate treatment are necessary, especially for the patients with risk factors.

